# Supplementary figures and images for: Recombinant origin and interspecies transmission of a HERV-K(HML-2)-related primate retrovirus with a novel RNA transport element
Source: eLife. 2024 Jul 22;13:e80216. doi: 10.7554/eLife.80216 (PMC11379458; doi:10.7554/eLife.80216)

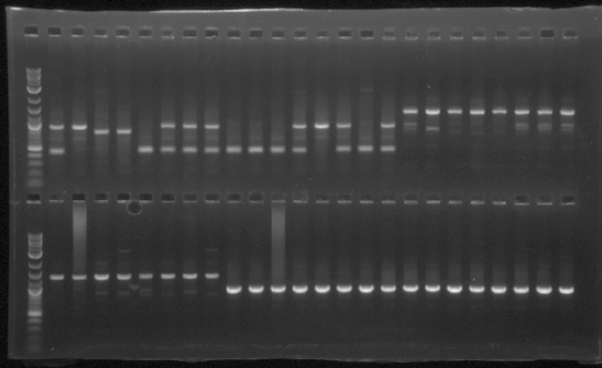

Supplement: Figure 3—figure supplement 1—source data 1. [file elife-80216-fig3-figsupp1-data1.zip › figure_3_supplement_1_source_data_1.png]

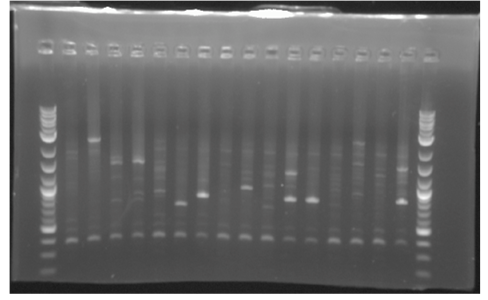

Supplement: Figure 3—figure supplement 1—source data 2. [file elife-80216-fig3-figsupp1-data2.zip › figure_3_supplement_1_source_data_2.png]

**B.**

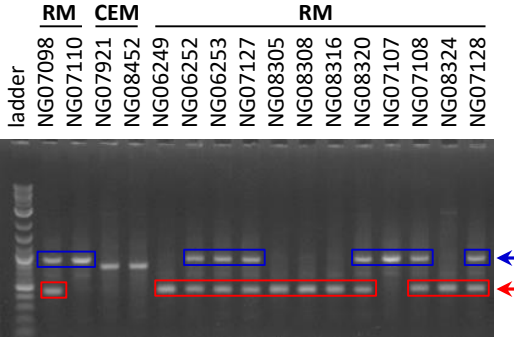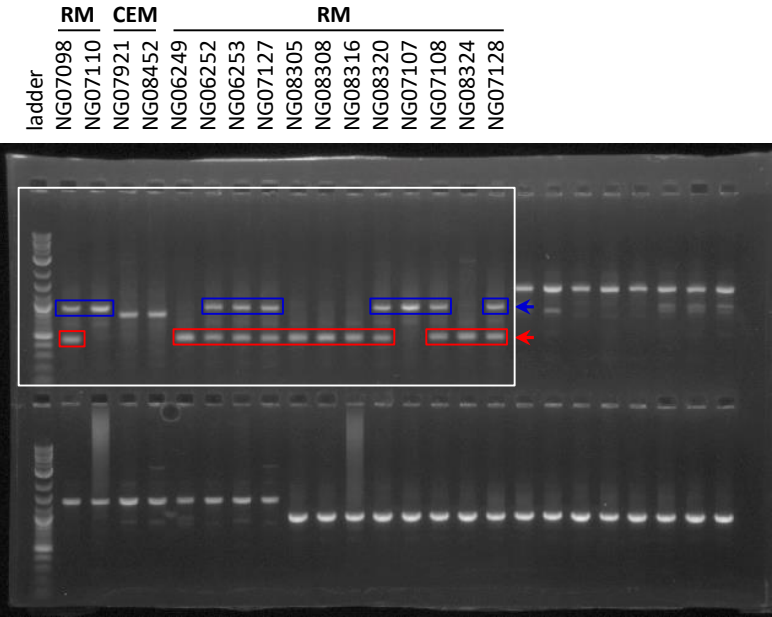

**Figure 3 supplement 1**

Supplement: Figure 3—figure supplement 1—source data 3. [file elife-80216-fig3-figsupp1-data3.zip › figure_3_supplement_1_source_data_3.pdf]

C.

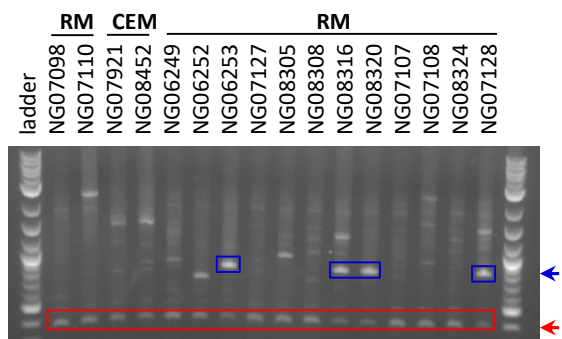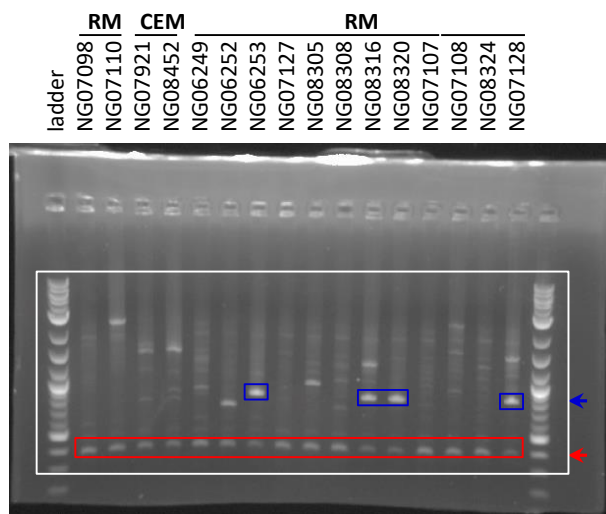

Figure 3 supplement 1

Supplement: Figure 3—figure supplement 1—source data 4. [file elife-80216-fig3-figsupp1-data4.zip › figure_3_supplement_1_source_data_4.pdf]
